# Analyzing the clinical profile and potential biomarkers to differentiate idiopathic angioedema from hereditary or mast cell-mediated angioedema^☆^

**DOI:** 10.1016/j.waojou.2026.101446

**Published:** 2026-08-26

**Authors:** Juan Liu, Xiangyi Cui, Nan Zhou, Yuxiang Zhi

**Affiliations:** Department of Allergy, Peking Union Medical College Hospital, Chinese Academy of Medical Sciences, Peking Union Medical College, Beijing Key Laboratory of Precision Medicine for Diagnosis and Treatment of Allergic Diseases, Beijing, China

**Keywords:** Angioedema, Hereditary, Mast cells, Biomarkers, Fibroblast activation protein alpha

## Abstract

**Purpose:**

Idiopathic angioedema (AE-UN) is a heterogeneous, exclusion-based clinical category rather than a single disease entity. The clinical characteristics and potential exploratory markers in patients currently classified as AE-UN remain poorly understood. This study aimed to evaluate clinical features and laboratory mediators in a selected cohort of patients fulfilling the current diagnostic definition of AE-UN.

**Methods:**

We compared 16 patients classified as AE-UN according to a stepwise diagnostic approach with 20 patients with hereditary angioedema (HAE), 20 patients with mast cell-mediated angioedema (AE-MC), and 24 healthy controls. AE-MC classification required recurrent angioedema with or without wheals, normal C4 and C1-inhibitor levels, and a convincing clinical response to mast cell-directed therapy, including H1-antihistamines up to four-fold standard dose when clinically indicated, corticosteroids, or standard-dose omalizumab (4–6 months). Patients without such a response and without confirmed HAE, drug-induced angioedema, or other recognized causes were classified as AE-UN. Baseline features, treatments, and laboratory factors were analyzed, including serum markers of inflammation, endothelial dysfunction, and serine proteases, as determined by ELISA.

**Results:**

AE-UN showed shorter disease duration compared to AE-MC and HAE (2.26 vs. 3.02 vs. 15.78, p < 0.0001), but experienced a relatively higher frequency of life-threatening attacks than AE-MC (25% vs. 10%). Meanwhile, facial edema was most common in AE-UN (68.75%). We did not find a significant difference regarding age and sex among all angioedema (AE) subtypes. IgG4 levels were increased in AE-UN rather than other AE groups, and demonstrated a statistical difference with HAE (p = 0.049). Besides, Ang-1, Ang-2, and Tie-2 were significantly elevated in all AE compared to controls. By contrast, the Ang-2/Ang-1 ratio was decreased in AE patients. Notably, in AE-UN compared to AE-MC and HAE, Fibroblast Activation Protein (FAP) concentration was significantly reduced, whereas for tPA and PLA2G7, the difference was not significant compared to controls and among AE subtypes.

**Conclusions:**

Lower FAP levels may represent an exploratory biomarker candidate in this selected AE-UN cohort, rather than a diagnostic marker for a homogeneous disease. Ang-1, Ang-2, and Tie-2 were increased across angioedema groups. Because of the small sample size, the exclusion-based nature of AE-UN, and the possibility of diagnostic reclassification, these findings should be interpreted as hypothesis-generating.

## Introduction

Angioedema (AE) is a non-itching, non-pitting swelling of the subcutaneous and/or submucosal tissues caused by increased vascular permeability. Recurrent angioedema can present with or without wheals.^1^^,^^2^ Bradykinin and/or histamine are known mediators that provoke AE attacks. However, a substantial part of angioedema remains unknown (42.5%),^3^ and 1 study from a large referral center showed that approximately one-third of their patients had idiopathic histaminergic angioedema.^4^ According to the latest Definition, acronyms, nomenclature, and classification of angioedema (DANCE) consensus,^5^ these patients are consequently diagnosed with idiopathic angioedema (AE-UN). Nevertheless, neither its pathophysiology has been elucidated, nor has the efficacy of therapy been defined,^6^ which results in a dilemma when acute AE-UN cases present at the emergency department.^7^ Further, the literature about AE-UN is currently limited, and reliable markers to differentiate AE-UN from hereditary angioedema (HAE), as well as from mast cell-mediated angioedema (AE-MC), are lacking.

In the present study, AE-UN is therefore used as a current clinical classification rather than as evidence of a unified pathophysiologic entity. This distinction is important because patients placed in the AE-UN category may later be reclassified as additional mast cell-mediated, bradykinin-mediated, genetic, or other mechanisms are identified.

Currently, the plasma enzymatic cascade system, inflammatory mediators, and endothelium-derived factors have been extensively reported in the mechanisms of HAE.^8^ However, only a few studies concentrate on exploring the major mediators in the pathogenesis of AE-UN. Mormile I et al^9^ assessed the levels of angiogenic and lymphangiogenic factors, complement components, inflammatory cytokines and chemokines, and activity of phospholipases A2 in the plasma of idiopathic non-histaminergic acquired angioedema patients, but they did not identify a certain biomarker, which indicated AE-UN represents a diagnostic challenge. Some reports have shown the successful response to omalizumab in AE-UN, suggesting the concomitance of a mast cell-mediated process.^10^ By contrast, since patients in AE-UN do not respond to antihistamines to some extent, an alternative mediator, such as bradykinin, may bring a new diagnostic perspective. One study found higher amounts of the vasoactive peptide bradykinin generated in AE-UN patients owing to contact system activator.^11^ This result demonstrated that the contact/kinin system is involved in the pathophysiology of some cases of AE-UN. Taken together, a more complex mechanism may underlie this specific type of angioedema.

Thus, our study aimed to describe the clinical characteristics of patients currently classified as AE-UN and to explore serum mediators that may help generate future mechanistic hypotheses. We analyzed components related to vascular integrity, serine proteases, and inflammation, while recognizing that AE-UN is not assumed to be biologically homogeneous and that any candidate marker requires validation in larger, well-characterized cohorts.

## Materials and methods

### Study design and patients

A case-control study was performed at the Peking Union Medical College Hospital (PUMCH). Fifty-six Chinese patients aged 18–70 years with recurrent angioedema were prospectively recruited from the outpatient clinic of the Allergology department between January 2023 and March 2025. Patients were classified into 3 groups through a stepwise diagnostic pathway: (1) HAE, type I or II; (2) AE-MC; and (3) AE-UN. HAE type I or II was diagnosed on the basis of compatible recurrent angioedema and reduced C1-inhibitor level and/or function with low C4. Diagnosis of AE-MC required all of the following: a history of recurrent angioedema with or without wheals; normal levels of C4 and C1 inhibitor; and a clear clinical response to mast cell-directed therapy, including H1-antihistamines up to four-fold standard dose when clinically indicated, corticosteroids, or standard-dose omalizumab (4–6 months). AE-UN was used as an exclusion-based clinical category according to the DANCE consensus. Patients were classified as AE-UN only when confirmed C1-inhibitor deficiency, ACE-inhibitor or other drug-induced angioedema, and other recognized causes had been excluded, and when the clinical course did not show a convincing response pattern consistent with mast cell-mediated disease. Family history, medication exposure, urticaria or wheals, attack locations, trigger factors, laboratory complement results, and treatment response were reviewed together to support the final clinical classification. The complete selection process is shown in Supporting Information: Figure S1. Twenty-4 healthy (HC) age- and sex-matched subjects without angioedema were enrolled as controls for serum biomarker levels. All patients signed written informed consent, and the study protocol was approved by the Ethics Committee of PUMCH (ethics approval number: K8599).

All the information about age, gender, allergic comorbidity, disease duration, concurrent urticaria, family history of angioedema, trigger factors, locations, emergency department visit, prodromal symptoms, and frequency of attacks was collected. Attack frequency was categorized as < 1 per year, ≥1 per year but <1 per month, ≥1 per month but <1 per week, ≥1 per week, and daily. We also recorded the prescribed treatment method, which was categorized as “none”, “antihistamine treatment”, “add-on treatment”, “danazol”, “Lanadelumab”, “Icatibant”, and “fresh frozen plasma (FFP)”.

In addition to baseline information, disease-specific laboratory parameters were assessed in our hospital's laboratory: C1–INH concentration (g/l), serum C3/C4 (g/l), Vascular Endothelial Growth Factor (VEGF, pg/ml), D-dimer (mg/l FEU), total IgE (kU/l), IgG4 (mg/l), Eosinophils (%), Eosinophils (10^9^/l), Antinuclear Antibody (ANA).

### Blood sampling and immunoassays for potential biomarkers

5 mL of venous blood sample was collected into the coagulation activator tube (BD Biosciences, Franklin Lakes, New Jersey, USA) after a routine diagnostic procedure, and then centrifuged at room temperature at 2000g for 20 min. The serum was divided into aliquots and kept frozen at −80 °C until further analysis. The blood samples used in this study were subjected to a single freeze-thaw cycle. All parameters were assessed in serum using commercially available enzyme-linked immunosorbent assay (ELISA) kits for Angiopoietin (Ang-) 1 and 2, fibroblast activation protein (FAP), E-selectin, tyrosine kinase with immunoglobulin and epidermal growth factor homology domains 2 (Tie-2), tissue plasminogen activator (tPA), and phospholipase A2 group VII (PLA2G7) according to the manufacturer's instructions (R&D Systems, Minneapolis, Minnesota, USA). The detection limits of ELISA are 62.5–4000 pg/ml for FAP, 78.1–5000 pg/ml for tPA, 94.7–23000 pg/ml for Ang-1 and Ang-2, 3500–850000 pg/ml for PLA2G7, 331–80000 pg/ml for E-Selectin, 609–150000 pg/ml for Tie-2.

### Statistical analysis

Data were analyzed by using the GraphPad Prism 9 software package (version 9.0, San Diego, CA, USA). Statistical comparison between the 2 groups was performed by an unpaired two-tailed *t*-test or two-tailed Mann-Whitney *U* test, whereas the one-way analysis of variance (ANOVA) or Kruskal-Wallis was used for comparison between 3 groups. The χ^2^ test was used to assess categorical variables. Results are presented as mean ± standard deviation (SD). A p-value less than or equal to 0.05 was described as a statistically significant difference.

## Results

### Demographics and clinical features

In total, 56 adult patients (63% female; mean age 39 years) confirmed to have recurrent AE were enrolled, clinical features and basic characteristics of the different subgroups were summarized in Table 1. There was no significant difference regarding age and sex. Patients with AE-MC were more likely to have comorbid allergic diseases (55% vs. 31.25% vs. 10%, p = 0.0096) and concurrent urticaria (70% vs. 18.75% vs. 5%, p < 0.0001) among the 3 AE types. Compared to the AE-MC and HAE groups, patients with AE-UN had the shortest disease course (2.26 vs. 3.02 vs. 15.78, p < 0.0001). For the positive family members with AE, as expected, HAE patients exhibited the highest proportion (p < 0.0001). The use of urgent care for at least an AE attack was seen in 26 (46.43%) out of 56 patients, but did not differ among these AE subtypes.

**Table 1:** Patient baseline characteristics.

Patient characteristics	AE-MC (n = 20)	AE-UN (n = 16)	HAE (n = 20)	p-value	Test
**Age,** mean (SD)	39.85 (16.89) 39.85 (16.89)	36.69 (15.44) 36.69 (15.44) 36.69 (15.44)	41.20 (12.98) 41.20 (12.98)	0.669 0.566 0.348	ANOVA t-test t-test
**Female,** n (%)	14 (70.00)	8 (50.00)	13 (65.00)	0.449	Chi-square
**Allergic comorbidity,** n (%)	11 (55.00)	5 (31.25)	2 (10.00)	**0.0096**	Chi-square
**Disease duration (years),** mean (SD)	3.02 (4.79) 3.02 (4.79)	2.26 (2.64) 2.26 (2.64) 2.26 (2.64)	15.78 (13.00) 15.78 (13.00)	**<0.0001** 0.578 **0.0003**	ANOVA t-test t-test
**Concurrent urticaria,** n (%)	14 (70.00)	3 (18.75)	1 (5.00)	**<0.0001**	Chi-square
**Family members with angioedema,** n (%)	0 (0.00)	0 (0.00)	18 (90.00)	**<0.0001**	Chi-square
**Emergency department visit,** n (%)	3 (15.00)	9 (56.25)	14 (70.00)		
**Trigger of attacks,** n (%)					
- Mental stress	1 (5.00)	0 (0.00)	6 (30.00)		
- Hormonal factors	0 (0.00)	0 (0.00)	4 (20.00)		
- External injury	2 (10.00)	1 (6.25)	3 (15.00)		
- Unknown	17 (85.00)	15 (93.75)	7 (35.00)		
**Correlation between the strength of prodromal symptoms and attacks,** n (%)					
- yes	1 (5.00)	0 (0.00)	6 (30.00)		
- no	19 (95.00)	16 (100.0)	14 (70.00)		
**Localization,** n (%)					
- Life-threatening: Uvula, throat, larynx	2 (10.00)	4 (25.00)	13 (65.00)		
- Face	12 (60.00)	11 (68.75)	10 (50.00)		
- Eyelid	8 (40.00)	8 (50.00)	3 (15.00)		
- Tongue	2 (10.00)	3 (18.75)	2 (10.00)		
- Lips	12 (60.00)	8 (50.00)	8 (40.00)		
- Genital area	2 (10.00)	2 (12.50)	7 (35.00)		
- Hands and feet	5 (25.00)	2 (12.50)	14 (70.00)		
- limbs	1 (5.00)	3 (18.75)	4 (20.00)		
- Spastic abdominal pain	2 (10.00)	3 (18.75)	15 (75.00)		
**Frequency of attacks**					
- <1 per year	0	0	1 (5.00)		
- ≥ 1 per year but <1 per month	3 (15.00)	6 (37.50)	8 (40.00)		
- ≥ 1 per month but <1 per week	14 (70.00)	7 (43.75)	8 (40.00)		
- ≥ 1 per week	3 (15.00)	2 (12.50)	3 (15.00)		
- Daily	0	1 (6.25)	0		

AE-MC: mast cell-mediated angioedema. AE-UN: idiopathic angioedema. HAE: hereditary angioedema. Bold numerical values indicate statistical significance.

We also investigated the triggers of the attack in 3 groups. The most commonly reported trigger factors were unknown (AE-MC: 85%, AE-UN: 93.75%, HAE: 35%), followed by external injury in AE-MC/AE-UN and mental stress in HAE. However, there was no correlation between the strength of prodromal symptoms and attacks of all 3 AE groups.

While in AE-UN, angioedema attacks most often occurred on the face (68.75%), followed by the eyelid and lips (50% respectively), up to 25% of AE-UN patients experienced life-threatening attacks, including the uvula, throat, and larynx. The hands, feet, and genital area were the least affected locations in this type (12.5%). In HAE, angioedema of the spastic abdominal pain was most frequent (75%), with 70% and 65% of patients suffering from the hands and feet, and uvula, throat, and larynx locations, respectively. The frequency of face angioedema was similar in AE-MC and AE-UN. Additionally, in AE-MC, angioedema of the face, as well as lips, occurred equally often (60%).

About attack frequency, most patients in these 3 different AE groups reported ≥1 per month but <1 per week (51.79%), only 1 patient of HAE presented with fewer than a single attack per year, while 1 patient showed daily attacks in the AE-UN group.

### Laboratory parameter analysis

Laboratory analyses revealed that patients with AE-UN exhibited elevated levels of IgG4 compared to HAE (p = 0.049), but with no significant difference compared to AE-MC patients (Table 2). D-dimer showed the lowest levels in the AE-UN angioedema types, although without a substantial difference. As expected, C1-inhibitor concentration (p = 0.0003) and C4 (p < 0.0001) were reduced only in HAE and significantly different between HAE and AE-UN (p = 0.0002). The results of VEGF were significantly higher in HAE compared to AE-MC (p < 0.0001) and AE-UN (p = 0.0008).

**Table 2:** Routine laboratory parameters.

Parameter	AE-MC (n = 20)	AE-UN (n = 16)	HAE (n = 20)	p-value	test
**C1-inhibitor concentration,** mean (SD), (norm: 0.21 - 0.39 g/l)	0.28 ± 0.05	0.30 ± 0.08	0.13 ± 0.16	**0.0003**	K-W∗
	0.28 ± 0.05	0.30 ± 0.08		0.518	MWU-test
	0.30 ± 0.08	0.13 ± 0.16	**0.0002**	MWU-test	
**C4,** mean (SD), (norm: 0.1 - 0.4 g/l)	0.25 ± 0.09	0.22 ± 0.08	0.06 ± 0.04	**< 0.0001**	K-W∗
	0.25 ± 0.09	0.22 ± 0.08		0.392	MWU-test
	0.22 ± 0.08	0.06 ± 0.04	**< 0.0001**	MWU-test	
**C3,** mean (SD), (norm: 0.73 - 1.46 g/l)	1.23 ± 0.25	1.17 ± 0.28	1.09 ± 0.25	0.153	K-W
	1.23 ± 0.25	1.17 ± 0.28		0.660	MWU-test
	1.17 ± 0.28	1.09 ± 0.25	0.397	MWU-test	
**VEGF,** mean (SD), (norm: ＜160 pg/ml)	72.27 ± 19.45	70.00 ± 29.59	169.20 ± 48.66	**< 0.0001**	K-W
	72.27 ± 19.45	70.00 ± 29.59		0.796	MWU-test
		70.00 ± 29.59	169.20 ± 48.66	**0.0008**	MWU-test
**D-Dimer,** mean (SD), (norm: 0 – 0.55 mg/l FEU)	1.05 ± 1.25	0.87 ± 1.84	2.32 ± 3.53	0.657	K-W
	1.05 ± 1.25	0.87 ± 1.84		0.580	MWU-test
		0.87 ± 1.84	2.32 ± 3.53	0.394	MWU-test
**total IgE,** mean (SD), (norm: 0 - 60 kU/l)	236.87 ± 238.51	231.94 ± 370.56	76.74 ± 87.06	0.272	K-W
**IgG4,** mean (SD), (norm: 36 - 2090 mg/l)	517.60 ± 316.07	717.11 ± 577.50	349.67 ± 261.01	0.130	K-W
	517.60 ± 316.07	717.11 ± 577.50		0.699	MWU-test
		717.11 ± 577.50	349.67 ± 261.01	**0.049**	MWU-test
**% Eosinophils,** mean (SD) (norm: 0.5 - 5.0 %)	1.95 ± 1.12	1.74 ± 1.36	1.36 ± 1.68	0.104	K-W
**Eosinophils abs.** mean (SD), (norm 0.02 - 0.5×10^9^/l)	0.13 ± 0.09	0.10 ± 0.08	0.08 ± 0.10	0.073	K-W
**ANA,** n (%)					
positive	2 (10.00)	2 (12.50)	2 (10.00)		
negative	18 (90.00)	14 (87.50)	18 (90.00)		

K-W∗: Kruskal-Wallis test. C4: Complement 4. C3: Complement 3. VEGF: Vascular Endothelial Growth Factor. ANA: Antinuclear Antibody. AE-MC: mast cell-mediated angioedema. AE-UN: idiopathic angioedema. HAE: hereditary angioedema. Bold numerical values indicate statistical significance.

No further statistically significant differences were detected regarding the levels of C3, total IgE, and Eosinophils. Furthermore, the difference in the positive or negative rate of ANA was also not significant among these AE types.

### Prescribed treatment strategy

Data on the prescribed treatment method are shown in Table 3. Add-on treatment was the most commonly used treatment for 56.25% of patients in the AE-UN group, and with statistical significance across all of the AE types (p = 0.011), glucocorticoids constituted the most frequently prescribed component of these combination regimens. Antihistamine monotherapy represented the second most common treatment strategy (50%), with 43.75% of AE-UN patients receiving 1–2 times the standard dosage. While it was the most prescribed therapy method in AE-MC (85%).

**Table 3:** Prescribed treatment method.

Maximum prescribed treatment	AE-MC (n = 20)	AE-UN (n = 16)	HAE (n = 20)	p-value	test
**None,** n (%)	0	1 (6.25)	0	0.280	Chi-square
**Antihistamine monotherapy,** n (%)	17 (85.00)	8 (50.00)	2 (10.00)	**＜0.0001**	Chi-square
1-2 fold dose	17 (85.00)	7 (43.75)	2 (10.00)		
3-＞4 fold dose	0	1 (6.25)	0		
**Add-on treatment,** n (%)	6 (30.00)	9 (56.25)	2 (10.00)	**0.011**	Chi-square
Montelukast	1 (5.00)	2 (12.5)	0		
Omalizumab	1 (5.00)	2 (12.50)	0		
Tranexamic acid	0	1 (6.25)	1 (5.00)		
Glucocorticoid	4 (20.00)	4 (25.00)	1 (5.00)		
**Danazol,** n (%)	0	0	9 (45.00)		
**Lanadelumab,** n (%)	0	0	1 (5.00)		
**Icatibant,** n (%)	0	2 (12.5)	6 (30.00)		
**FFP,** n (%)	0	2 (12.5)	0		

FFP: Fresh frozen plasma. AE-MC: mast cell-mediated angioedema. AE-UN: idiopathic angioedema. HAE: hereditary angioedema. Bold numerical values indicate statistical significance.

Notably, there were 2 patients in the AE-UN group reported to be treated with Icatibant and FFP, respectively, as well as the use of omalizumab. Among HAE patients, danazol was the most frequently administered treatment (45%), followed by Icatibant in 6 cases (30%).

### Potential serum biomarker immunoassays

**Fig. 1:**
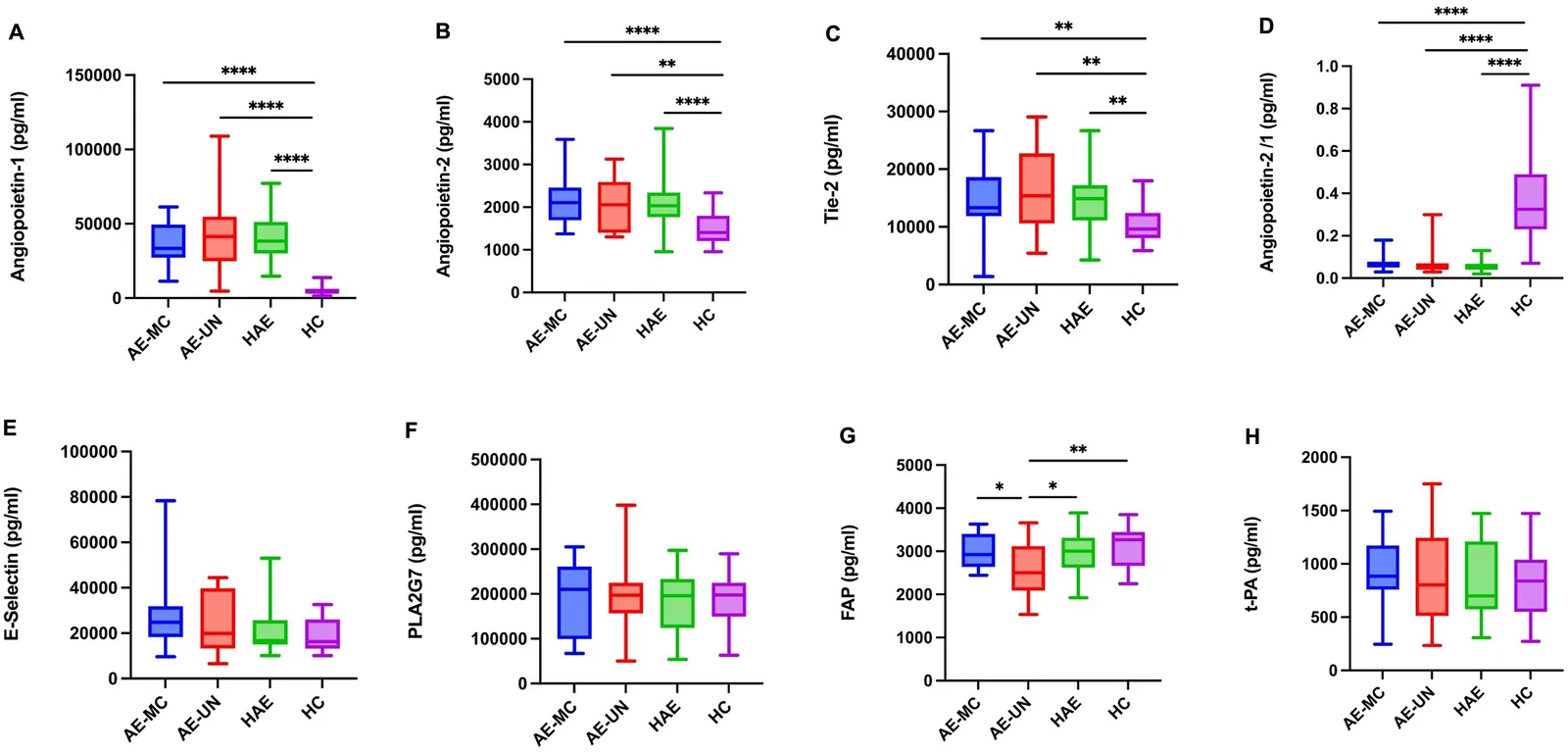
Serum levels of Ang-1 (A), Ang-2 (B), Tie-2 (C), Ang-2/Ang-1 (D), E-Selectin (E), PLA2G7 (F), FAP (G), t-PA (H) in healthy controls and different angioedema subtypes. AE-MC (n = 20), AE-UN (n = 16), HAE (n = 20), HC (n = 24). Values in the box plots are shown in pg/ml. The horizontal line inside the box represents the median, and the bottom and top boundaries of the boxes are the 25th and 75th percentiles. The whiskers represent the 5th and 95th percentiles. ∗p < 0.05, ∗∗p < 0.01, ∗∗∗p < 0.001, ∗∗∗∗p < 0.0001 as assessed by MWU-test

We detected the concentration of different serum factors in all 3 AE groups, including endothelial dysfunction parameters, serine proteases, and proinflammatory mediators. As can be seen in Fig. 1A and B and Table 4, the levels of serum Ang-1 and Ang-2 in all AE subtypes were significantly increased compared to HC patients. However, there was no difference among AE-UN, AE-MC, and HAE. Similarly, angiopoietin receptor Tie-2 concentration was higher in all the AE patients than in healthy controls (Fig. 1C). But the Ang-2/Ang-1 ratio was decreased in all the patients versus healthy controls (Fig. 1D). Further parameter results were obtained when we observed that the E-selectin level in different AE types was comparable to the HC group (Fig. 1E).

**Table 4:** Differences of these serum proteins between AE-MC, AE-UN, HAE, and healthy controls.

Parameters (pg/ml)	AE-MC (n = 20)	AE-UN (n = 16)	HAE (n = 20)	HC (n = 24)	p-value	Test
Angiopoietin-2	2166.10 ± 565.41	2079 ± 598.97	2084.89 ± 559.91	1498.00 ± 352.83	**<0.0001**	K–W∗
Angiopoietin-1	36755.35 ± 13580.55	43216.25 ± 24602.85	40775.35 ± 17713.88	5318.46 ± 3181.37	**<0.0001**	K–W
Tie-2	14672.60 ± 6135.16	16271.81 ± 7291.98	14557.85 ± 5426.15	10526.08 ± 3027.20	**0.005**	K–W
E-selectin	29208.10 ± 17632.19	24124.75 ± 13117.83	20587.90 ± 9469.46	19628.54 ± 7094.27	0.231	K–W
PLA2G7	190665.60 ± 81626.25	195650.06 ± 82892.52	180362.55 ± 66513.17	185413.13 ± 57157.61	0.936	K–W
FAP	3000.57 ± 402.19	2585.96 ± 573.14	3007.76 ± 497.95	3110.31 ± 477.24	**0.025**	K–W
t-PA	894.61 ± 326.48	849.56 ± 447.71	823.47 ± 350.52	819.43 ± 354.68	0.874	K–W

K–W∗: Kruskal-Wallis test. AE-MC: mast cell-mediated angioedema. AE-UN: idiopathic angioedema. HAE: hereditary angioedema. HC: healthy control. Tie-2: tyrosine kinase with immunoglobulin and epidermal growth factor homology domains 2. PLA2G7: phospholipase A2 group VII. FAP: fibroblast activation protein. t-PA: tissue plasminogen activator. Bold numerical values indicate statistical significance.

Next, we measured the serum levels of proinflammatory mediators in patients with AE and healthy controls. PLA2G7 levels of the 3 AE subtypes were not different from those of HC patients (Fig. 1F).

The most remarkable exploratory difference in our study was observed for FAP (Fig. 1G). FAP levels in the AE-UN group were significantly lower than in the AE-MC (p = 0.034) and HAE groups (p = 0.030). Therefore, FAP should be considered an exploratory biomarker candidate in this selected AE-UN cohort, not a diagnostic marker for AE-UN as a homogeneous disease. In addition, FAP concentration was decreased in AE-UN versus healthy controls (p = 0.003). However, we did not find a significant difference in FAP levels between the AE-MC and HAE groups (p = 0.890). Moreover, age, sex, disease duration, IgG4 levels, and VEGF concentration in patients with AE-UN did not correlate with FAP levels (data not shown). Fig. 1H showed that tPA levels in each AE type were similar to those of controls, without statistical significance.

## Discussion

To our knowledge, this is the first study in China to focus on the clinical and laboratory profile of patients currently classified as AE-UN, an exclusion-based and heterogeneous clinical category. The main findings of the study are: (1) the disease duration of AE-UN was the shortest, and 25% of patients experienced life-threatening attacks; (2) IgG4 levels in AE-UN patients were higher than in the other 2 angioedema groups; and (3) FAP was lower in this selected AE-UN cohort than in AE-MC and HAE, supporting its further evaluation as a hypothesis-generating biomarker candidate, while Ang-1, Ang-2, and Tie-2 appeared to be increased across angioedema groups.

The baseline demographics and clinical characteristics were in keeping with previously published research investigating AE-UN patients across the world to a certain degree.^12^^,^^13^ For both AE-MC and HAE, patients were predominantly women, but AE-UN showed an equal gender ratio and had the shortest disease duration. A recent retrospective study showed that AE-UN without wheals were older and experienced more often a swelling of the tongue compared to patients with wheals (50% vs. 33%).^14^ However, about 25% of patients in our AE-UN experienced life-threatening attacks, potential explanations include both the limited sample size and our omission of wheal-based subgroup analyses. The most common site affected was the face, which line with previous study.^15^ For the laboratory factors, we surprisingly found an elevation of IgG4 in AE-UN rather than AE-MC and HAE patients, but it is questionable whether this specific phenomenon can be explained by a specific mechanism in patients of AE-UN. Reviewing the literature, we did not find evidence for the role of IgG4 in idiopathic angioedema. Taken together, no distinct diagnosis relationship was found for AE-UN versus AE-MC or HAE, although some clinical characteristics were more frequently related to a certain angioedema type, no diagnostic clue existed. We need to design a study that includes regular prospective follow-up visits and blood collection in the future.

The landscape of therapeutic methods has significantly widened for AE-MC and HAE in recent years, but currently, there is a lack of consensus in the therapeutic approaches for this unknown form of angioedema, although a study conducted by Adil A et al^16^ demonstrated 74% of AE-UN patients in Canada received long-term prophylaxis, including subcutaneous pdC1-INH and tranexamic acid. Other authors also reported the efficacy of omalizumab^17^^,^^18^ and Lanadelumab^19^ in AE-UN. Our study was not designed to evaluate the efficacy of treatment because of the lack of long-term follow-up. Interestingly, we analyzed the patients’ maximum prescribed therapeutic methods, and most of them experienced add-on treatment, like an up-dose antihistamine, omalizumab, Icatibant, and FFP. From an immunomodulatory perspective, cyclosporin A is also worth noting, as it inhibits calcineurin-dependent T-cell activation and has been used in selected immune-mediated diseases. Filaci et al^20^ reported long-term cyclosporin A treatment in systemic sclerosis, suggesting that this therapeutic strategy may be relevant in chronic immune-driven conditions. However, cyclosporin A was not evaluated in our cohort, and its potential role in AE-UN remains speculative. Controlled studies would be required before any conclusion could be drawn regarding its efficacy or safety in this setting. Overall, these research data indicated that bradykinin or histamine mediators might be involved in the pathogenesis of the disease, but the underlying mechanisms in AE-UN largely remain to be elucidated.

In addition to bradykinin- and mast cell-related mechanisms, immune regulatory pathways may also contribute to the heterogeneity of AE-UN. The immune background of recurrent angioedema may extend beyond the endothelial and protease-related markers evaluated in the present study. Vitamin D is increasingly regarded as an immune-active hormone rather than only a regulator of calcium metabolism, because its receptor is expressed by several immune-cell populations and its downstream signaling can influence antigen presentation, T-cell polarization, regulatory T-cell activity, B-cell differentiation, and epithelial antimicrobial defense.^21^^,^^22^ In parallel, the intestinal microbiota can shape mucosal and systemic immunity through microbial metabolites, barrier regulation, and effects on inflammatory-cell activation. Recent reviews by Murdaca and colleagues^21^ proposed that alterations in vitamin D status and microbiome composition may contribute to the immune imbalance observed in autoimmune and allergic diseases, with sex and age acting as additional modifiers. We did not assess serum vitamin D concentrations or microbiome composition in this cohort; therefore, these pathways cannot be directly linked to our biomarker findings. Nevertheless, they provide a biologically plausible framework for future studies exploring why AE-UN patients differ in allergic comorbidity, treatment response, and inflammatory profiles. Prospective work combining vitamin D assessment, microbiome analysis, mediator profiling, and genetic testing may help determine whether these factors define clinically meaningful AE-UN endotypes.

In our study, to gain a better understanding of the possible involvement of mediators in the pathogenesis of AE-UN, we focused on 3 different serum parameters related to the dysfunction of the vascular system, including endothelial dysfunction parameters (Ang-1, Ang-2, Tie-2, E-selectin), serine proteases (FAP, tPA), and proinflammatory mediators (PLA2G7). We confirmed that both the levels of Ang-1 and Ang-2, which facilitate vascular permeability, were significantly higher in all AE patients than in the controls. Meanwhile, the Ang-2/Ang-1 ratio was decreased among these angioedema patients. Our data is similar to previously published results, which show increased Ang-2 levels in HAE with more frequent attacks.^23^ Conversely, another study found that Ang-1 decreased in AE-UN compared to controls, while Ang-2 levels were similar to those in controls.^9^ These contrasting observations might be due to the greater heterogeneity of AE-UN, and they underwent the procedure during remission in their research.

Tie-2, a receptor tyrosine kinase that is almost exclusively expressed in endothelial cells, plays a vital role in vascular stability.^24^ Ang-1 and Ang-2 act as agonistic and antagonistic ligands of Tie-2 signaling separately.^25^ A previous study reported that Tie-2 was specifically increased in HAE, and its level correlated significantly with C4, C1–INH activity, and C1–INH function.^26^ Interestingly, this was the first study to investigate Tie-2 in angioedema type. At the same time, increased Tie-2 levels in AE-MC, AE-UN, and HAE groups compared to the HC groups were observed in our study, which further confirmed that Tie-2 may participate in the pathogenesis of angioedema.

Nevertheless, analysis of E-selectin levels failed to discriminate between AE-UN and other angioedema subtypes, and did not show an upregulation in any other angioedema patients. E-selectin is an important and early marker of endothelial activation and also inflammation.^27^ The expression of E-selectin is mainly assessed in the HAE cohort, which demonstrated a negative or positive correlation between C1–INH and E-selectin,^26^^,^^28^^,^^29^ moreover, they even suggested that E-selectin was increased in bradykinin-mediated AE (BK-AE) only.

We also did not find striking abnormalities regarding PLA2G7 between AE patients and healthy controls. The PLA2 family can directly modulate the permeability of vascular endothelial cells,^23^ and PLA2G7 has been demonstrated to be associated with the activation and migration of macrophages, subsequently inducing the production of inflammatory cytokines.^30^ Thus, a study found that AE-UN patients have higher activity of PLA2,^9^ the mechanism underlying this different observation is still unclear.

The most intriguing finding is that the FAP may be an exploratory biomarker candidate in this selected AE-UN cohort. FAP is a type II transmembrane serine protease, which is related to wound healing, chronic fibrosing diseases, autoimmune diseases, and deep vein thrombosis.^31^ A study conducted in 2023 gained significance, showing that an increased thromboinflammatory load in HAE,^32^ and Bindke G et al^26^ showed an overexpression of FAP in AE-MC and BK-AE types rather than in healthy controls, suggesting that FAP may reflect angioedema-related biology but may not be specific to a single subtype. In our study, AE-UN patients had the lowest FAP levels compared to the AE-MC and HAE patients, as well as the concentration of D-dimer, but no direct correlation between FAP and D-dimer in AE-UN (data not shown). Because AE-UN is heterogeneous and exclusion-based, these data should be interpreted cautiously and should not be used to define AE-UN as a uniform disease or to exclude occult HAE with normal C1 esterase inhibitor (HAE-nC1INH) or mast cell-mediated mechanisms in individual patients. On the other hand, about the expression of tPA, we failed to find statistical significance between AE patients and healthy controls, since it is released from endothelial cells by bradykinin itself, and shear stress.^33^ It is supposed that tPA is more likely associated with the bradykinin-forming system.

We are aware that this study had limitations. First, the small sample size and single-center design are the main limitations due to AE-UN and HAE being rare diseases. Second, AE-UN should not be interpreted as a single homogeneous disease entity. It is an exclusion-based clinical category, and patients currently classified as AE-UN may include heterogeneous mechanisms that could be reclassified as knowledge advances. Third, a key challenge in this field lies in distinguishing AE-UN from HAE-nC1INH, especially because the absence of family history alone cannot exclude HAE, given incomplete penetrance and asymptomatic mutation carriers. Based on recent international studies,^34^^,^^35^ AE-UN–apart from lacking causative gene mutations and having normal C1 esterase inhibitor levels–typically presents with no family history of angioedema and later disease onset. In line with this, all AE-UN patients enrolled in our study had no family history. However, comprehensive genetic testing was not performed in all AE-UN patients. Whole-genome sequencing (WGS) was performed only in 5 patients who were clinically suspected of HAE-nC1INH, and no pathogenic or likely pathogenic variants explaining their clinical phenotype were identified (data not shown). Therefore, genetically mediated HAE cannot be completely excluded in the remaining patients, and some degree of diagnostic misclassification remains possible. Future multicenter studies incorporating comprehensive genetic testing in all AE-UN patients will be essential to further validate our findings. Fourth, although AE-MC was defined by clinical response to mast cell-directed therapy, it is impossible to exclude mast cell involvement with absolute certainty in every patient currently classified as AE-UN. Future multicenter studies incorporating comprehensive genetic testing, standardized therapeutic response assessment, and longitudinal follow-up will be essential to validate whether FAP has clinical utility as an exploratory distinguishing parameter. Fifth, the enrolled cases were not systematically classified by disease phase (active attack or clinical remission), potentially leading to an incomplete assessment of endothelial characteristics.

In conclusion, this study compared clinical and laboratory features among patients with AE-UN, AE-MC, and HAE in a Chinese cohort. Decreased FAP may serve as a promising exploratory biomarker candidate in this selected AE-UN cohort, while Ang-1, Ang-2, and Tie-2 may represent general markers of angioedema. However, AE-UN remains a heterogeneous exclusion-based category, and these findings should not be interpreted as establishing a homogeneous disease entity or a definitive diagnostic biomarker. Larger multicenter studies with comprehensive genetic testing and longitudinal follow-up are needed before these observations can be translated into clinical practice.

## Availability of data and materials

The datasets analyzed during the current study are available from the corresponding author on reasonable request.

## Authors’ consent for publication

All authors have approved the final manuscript and approved of the submission to World Allergy Organization Journal.

## Author contribution statement

Juan Liu contributed to the design of the study, and drafted the manuscript. Juan Liu and Xiangyi Cui performed the data analysis. Xiangyi Cui and Nan Zhou are responsible for sample collection. Yuxiang Zhi designed the experiments and provided funding. All authors have contributed to the manuscript and approved the submitted version. ^#^ These authors have contributed equally to this work and share first authorship.

## Ethical approval and statement

This study was in accordance with the Declaration of Helsinki and approved by the Ethics Review Committee of Peking Union Medical College Hospital, with registration number (K8599). Informed consent was obtained from all participants.

## Disclosure statement of generative artificial intelligence (AI) and AI-assisted technologies

Nothing to disclose.

## Funding

This study was supported by the 10.13039/501100001809National Natural Science Foundation of China (B063220), the National High-Level Hospital Clinical Research Funding, 10.13039/501100008235Peking Union Medical College Hospital (2025-PUMCH-A-002), and the Fundamental Research Funds for the Central Universities, 10.13039/501100011176Peking Union Medical College (3332024108).

## Conflict of interest

All authors have nothing to declare.

## References

[1] Belbézier A., Bocquet A., Bouillet L. (2020). Idiopathic angioedema: current challenges. J Asthma Allergy.

[2] Gülbahar O. (2021). Angioedema without wheals: a clinical update. Balkan Med J.

[3] Pall A.H., Lomholt A.F., von Buchwald C., Bygum A., Rasmussen E.R. (2020). Clinical features and disease course of primary angioedema patients in a tertiary care hospital. J Asthma Allergy.

[4] Zingale L.C., Beltrami L., Zanichelli A. (2006). Angioedema without urticaria: a large clinical survey. CMAJ (Can Med Assoc J).

[5] Reshef A., Buttgereit T., Betschel S.D. (2024). Definition, acronyms, nomenclature, and classification of angioedema (DANCE): AAAAI, ACAAI, ACARE, and APAAACI DANCE consensus. J Allergy Clin Immunol.

[6] Kesh S., Bernstein J.A. (2022). Isolated angioedema: a review of classification and update on management. Ann Allergy Asthma Immunol.

[7] Hébert J., Boursiquot J.N., Chapdelaine H. (2022). Bradykinin-induced angioedema in the emergency department. Int J Emerg Med.

[8] Porebski G., Kwitniewski M., Reshef A. (2021). Biomarkers in hereditary angioedema. Clin Rev Allergy Immunol.

[9] Mormile I., Gigliotti M.C., Ferrara A.L. (2024). Clinical features and potential markers of disease in idiopathic non-histaminergic angioedema, a real-life study. Immunol Res.

[10] Casale T.B., Trzaskoma B., Holden M., Bernstein J.A., Maurer M. (2024). Does angioedema in patients with chronic spontaneous urticaria impact response to omalizumab?. World Allergy Organ J.

[11] Suffritti C., Calzari P., Depetri F. (2024). Contact system activation and bradykinin generation in patients with idiopathic angioedema. J Investig Allergol Clin Immunol.

[12] Schulkes K.J.G., Van den Elzen M.T., Hack E.C. (2015). Clinical similarities among bradykinin-mediated and mast cell-mediated subtypes of non-hereditary angioedema: a retrospective study. Clin Transl Allergy.

[13] Nettis E., Di Leo E., Racanelli V., Macchia L., Vacca A. (2019). Idiopathic nonhistaminergic angioedema: a single-center real-life experience from Italy. Allergy.

[14] Soegiharto R., Alizadeh Aghdam M., Knulst A.C., Röckmann H. (2023). Clinical profile of idiopathic angioedema based on severity and treatment response is independent of the presence of concomitant wheals. JEADV Clinical Practice.

[15] Rosenberg D.L., Mathur S.K., Viswanathan R.K. (2019). Characterization of idiopathic angioedema in a university-based allergy/immunology practice. Ann Allergy Asthma Immunol.

[16] Adatia A., Boursiquot J.N., Goodyear D. (2024). Real-world outcomes of patients with hereditary angioedema with normal C1-inhibitor function and patients with idiopathic angioedema of unknown etiology in Canada. Allergy Asthma Clin Immunol.

[17] Faisant C., Du Thanh A., Mansard C. (2017). Idiopathic non-histaminergic angioedema: successful treatment with omalizumab in five patients. J Clin Immunol.

[18] Brunetta E., Shiffer D., Folci M. (2018). Omalizumab for idiopathic nonhistaminergic angioedema: evidence for efficacy in 2 patients. Case Reports Immunol.

[19] Gamboa P., Galán C., Arrien A., Segurola A., Jáuregui I. (2024). Life-threatening idiopathic nonhistaminergic acquired Angioedema with response to Lanadelumab. J Investig Allergol Clin Immunol.

[20] Filaci G., Cutolo M., Basso M. (2001). Long-term treatment of patients affected by systemic sclerosis with cyclosporin A. Rheumatology.

[21] Murdaca G., Tagliafico L., Page E., Paladin F., Gangemi S. (2024). Gender differences in the interplay between vitamin D and microbiota in allergic and autoimmune diseases. Biomedicines.

[22] Murdaca G., Gerosa A., Paladin F. (2021). Vitamin D and microbiota: is there a link with allergies?. Int J Mol Sci.

[23] Ferrara A.L., Bova M., Petraroli A. (2020). Hereditary angioedema attack: what happens to vasoactive mediators?. Int Immunopharmacol.

[24] Pham A.V.Q., Na Y., Suk G. (2025). Identification of Tie2 as a sensor for reactive oxygen species and its therapeutic implication. Redox Biol.

[25] Jo G., Bae J., Hong H.J. (2021). Structural insights into the clustering and activation of Tie2 receptor mediated by Tie2 agonistic antibody. Nat Commun.

[26] Bindke G., Gehring M., Wieczorek D. (2022). Identification of novel biomarkers to distinguish bradykinin-mediated angioedema from mast cell-/histamine-mediated angioedema. Allergy.

[27] Waclawovsky A.J., Dos Santos E.B., de Oliveira A.A.R., Stubbs B., Schuch F.B. (2025). Plasma biomarkers of endothelial function in people with depressive disorder: a systematic review and meta-analysis. J Affect Disord.

[28] Czúcz J., Schaffer G., Csuka D. (2012). Endothelial cell function in patients with hereditary angioedema: elevated soluble E-selectin level during inter-attack periods. J Clin Immunol.

[29] Cai S., Davis A.E. (2003). 3rd. Complement regulatory protein C1 inhibitor binds to selectins and interferes with endothelial-leukocyte adhesion. J Immunol.

[30] Jin L., Jiang M., Qian J. (2024). The role of lipoprotein-associated phospholipase A2 in inflammatory response and macrophage infiltration in sepsis and the regulatory mechanisms. Funct Integr Genomics.

[31] Oguri N., Gi T., Nakamura E. (2024). Expression of fibroblast activation protein-α in human deep vein thrombosis. Thromb Res.

[32] Gramstad O.R., Schjalm C., Mollnes T.E., Nielsen E.W. (2023). Increased thromboinflammatory load in hereditary angioedema. Clin Exp Immunol.

[33] Napolitano F., Montuori N. (2021). The role of the plasminogen activation System in angioedema: novel insights on the pathogenesis. J Clin Med.

[34] Christiansen S.C., Banerji A., Bernstein J.A. (2025). Hereditary angioedema with normal C1 inhibitor: a quarter century of forward progress and persisting obstacles. J Allergy Clin Immunol Pract.

[35] Busse P.J., Christiansen S.C. (2020). Hereditary angioedema. N Engl J Med.

