# Supplementary material for: Analyzing the clinical profile and potential biomarkers to differentiate idiopathic angioedema from hereditary or mast cell-mediated angioedema
Source: World Allergy Organ J. 2026 Aug 26;19(9):101446. doi: 10.1016/j.waojou.2026.101446 (PMC13544275; doi:10.1016/j.waojou.2026.101446)
Supplement: Supplementary file 1 [file mmc2.pdf]

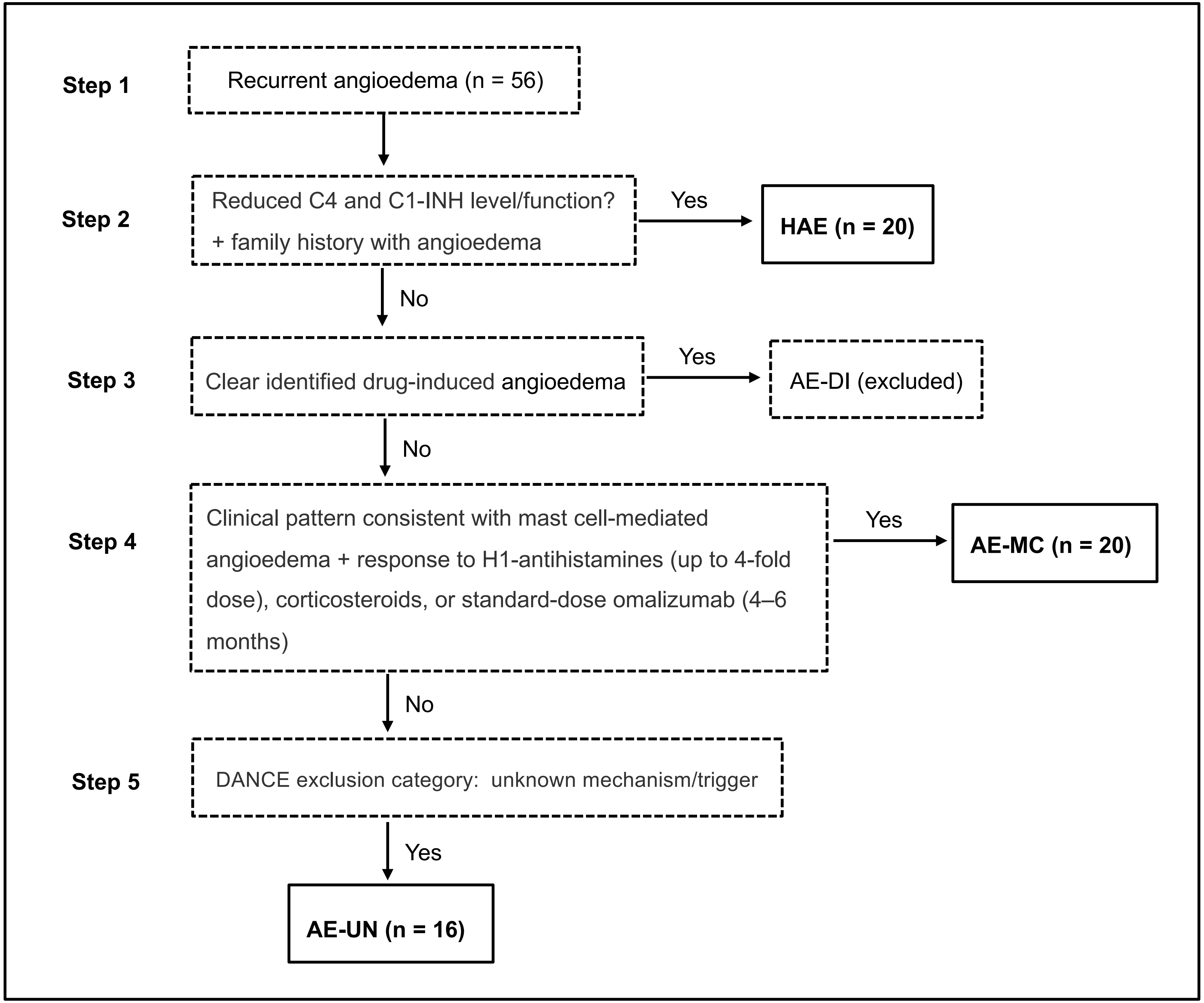

Step 1
Recurrent angioedema (n = 56)
Step 2
Reduced C4 and C1-INH level/function?
+ family history with angioedema
Yes
HAE (n = 20)
No
Step 3
Clear identified drug-induced angioedema
Yes
AE-DI (excluded)
No
Step 4
Clinical pattern consistent with mast cell-mediated angioedema + response to H1-antihistamines (up to 4-fold dose), corticosteroids, or standard-dose omalizumab (4–6 months)
Yes
AE-MC (n = 20)
No
Step 5
DANCE exclusion category: unknown mechanism/trigger
Yes
AE-UN (n = 16)
