# Supplementary material for: Analyzing the clinical profile and potential biomarkers to differentiate idiopathic angioedema from hereditary or mast cell-mediated angioedema
Source: World Allergy Organ J. 2026 Aug 26;19(9):101446. doi: 10.1016/j.waojou.2026.101446 (PMC13544275; doi:10.1016/j.waojou.2026.101446)
Supplement: Supplementary file 2 [file mmc1.docx]

**Supplementary Figure S1. Flowchart for the classification of patients with recurrent angioedema.** A total of 56 patients with recurrent angioedema were classified through a stepwise assessment of complement parameters, family history, potential drug-related causes, clinical phenotype, and treatment response. Patients meeting the criteria for hereditary angioedema were classified as HAE (n = 20). Patients with a clearly identified drug-related cause were classified as drug-induced angioedema (AE-DI) and excluded. Patients with a clinical pattern consistent with mast cell-mediated angioedema and a response to H1-antihistamines at up to fourfold the standard dose, corticosteroids, or standard-dose omalizumab administered for 4–6 months were classified as AE-MC (n = 20). Patients whose underlying mechanism or trigger remained unknown after exclusion of these categories were classified as AE-UN (n = 16).

*Abbreviations:* AE-DI, drug-induced angioedema; AE-MC, mast cell-mediated angioedema; AE-UN, idiopathic angioedema; C1-INH, C1 inhibitor; C4, complement component 4; DANCE, Definition, Acronyms, Nomenclature, and Classification of Angioedema; HAE, hereditary angioedema.
